# Left colic artery diameter is an important factor affecting anastomotic blood supply in sigmoid colon cancer or rectal cancer surgery: a pilot study

**DOI:** 10.1186/s12957-022-02774-0

**Published:** 2022-09-27

**Authors:** Bo Li, Jianan Wang, Shaohui Yang, Jie Shen, Qi Li, Qiqi Zhu, Wei Cui

**Affiliations:** Department of Colorectal Surgery, Ningbo Medical Centre Lihuili Hospital, Ning Bo, 315000 China

**Keywords:** Left colic artery, Colorectal surgery, Anastomotic blood supply, Stump pressure

## Abstract

**Background:**

Anastomotic blood supply is vital to anastomotic healing. The aim of this study was to demonstrate the effect of the left colic artery (LCA) on blood supply in the anastomotic area, explore the relationship between individual differences in the LCA and blood supply in the anastomotic area, and elucidate the relevant indications for LCA retention during radical resection for sigmoid or rectal cancer.

**Method:**

Radical sigmoid or rectal cancer resection with LCA retention was performed in 40 patients with colorectal cancer who participated in this study. Systemic pressure, LCA diameter, and the distance from the root of the LCA to the root of the inferior mesenteric artery were measured and recorded. The marginal artery stump pressure in the anastomotic colon before and after the LCA clamping was measured, respectively.

**Results:**

There is a significant difference between the marginal artery stump pressure before LCA ligation and after ligation (53.1 ± 12.38 vs 42.76 ± 12.71, *p* < 0.001). The anastomotic blood supply positively and linearly correlated with body mass index and systemic pressure. Receiver-operating curve analysis revealed that LCA diameter (area under the curve 0.971, cutoff 1.95 mm) was an effective predictor of LCA improving anastomosis blood supply. No relationship was found between the LCA root location and anastomotic blood supply.

**Conclusion:**

Preserving the LCA is effective in improving blood supply in the anastomotic area, and larger LCA diameters result in a better blood supply to the anastomotic area.

**Supplementary Information:**

The online version contains supplementary material available at 10.1186/s12957-022-02774-0.

## Introduction

One of the most serious complications of colorectal cancer surgery is anastomotic leakage. It occurs in 1 to 30% of all cases [1–4], with the highest rate (of up to 20%) reported for rectal anastomotic leakage [5]. An anastomotic fistula seriously affects patients’ quality of life, prognosis, and hospitalization costs and increases the risk of death [6, 7]. It is well known that there are many risk factors can cause the occurrence of postoperative anastomotic leaks, such as male gender, preoperative radiotherapy, and a low rectal anastomosis [8]. And the intraoperative anastomotic status is one of factors highly correlated with the occurrence of postoperative anastomotic leaks, including anastomosis blood supply, anastomotic tone, and anastomotic integrity, of which anastomotic blood supply is particularly important. Currently, high ligation of the inferior mesenteric artery (IMA) results in more thorough lymph node dissection and simpler surgical procedures and is the mainstream modality for radical sigmoid or rectal cancer surgery. After high ligation of the IMA, the marginal artery of the middle colic artery supplies most of the blood to the distal intestinal canal. There is still no consensus as to whether retaining the left colic artery (LCA) can reduce the incidence of anastomotic complications [9]. Studies have shown that radical sigmoid or rectal cancer resection that preserves the LCA can effectively increase blood supply in the anastomotic area, thereby reducing the incidence of postoperative anastomotic leakage [10]. In addition, studies also have shown that in either IMA or LCA clamping, there was a significant reduction in colonic blood flow at the proximal site of the anastomosis [11, 12]. However, it has also been shown that retaining the LCA does not help either reduce the incidence of postoperative anastomotic leakage [13] or increase blood supply in the anastomotic area [14].

The IMA and its branches have large individual differences, for example, the origin of the LCA can be 15 to 88 mm from the root of the IMA [15, 16]. There is a clear correlation between the diameter of IMA and the stage of rectal cancer, and the diameter of the IMA significantly increases from stage I to stage II and from stage II to stage III of rectal cancer [17]. In addition, there is a large variation in the track of the LCA, with 53.2% of the artery traveling up the cephalic side to the splenic flexure of the transverse colon and 27.1% traveling the upper left and diagonally to the upper part of the descending colon [18]. Therefore, to clarify the indications for LCA retention during radical resection of sigmoid or rectal cancer, it is necessary to explore the correlation between individual differences in the LCA and blood supply in the anastomotic area.

Invasive arterial pressure measurement is the direct determination of intravascular pressure, which is the “gold standard” for blood pressure measurement, and can reflect pressure changes in the arteries in a timely and accurate manner and objectively reflect blood supply. In this study, we measured the marginal artery stump pressure (MASP) in the anastomotic colon to reflect the blood supply of the anastomotic area, explore the correlation between individual differences in the LCA and blood supply in the anastomotic area, with an aim to determine the relevant indications for LCA retention during radical resection of sigmoid or rectal cancer, and provide a theoretical basis for developing a personalized surgical protocol for colorectal cancer.

## Patients and methods

### Patient screening

This study was approved by the Institutional Review Board of Ningbo Medical Center Li Huili Hospital. Patients consecutively recruited from February 2022 to June 2022 with sigmoid or rectal cancer diagnosed by pathological biopsy, colonoscopy, and enhanced computed tomography were treated at the Department of Colorectal Anorectal Surgery of the Ningbo Medical Center Li Huili Hospital. This study considered only solitary radical resectable sigmoid or rectal cancers 5–25 cm from the anus. The inclusion criteria were as follows: (1) patients with sigmoid or rectal cancer who required surgery; (2) patients aged > 18 years and ≤ 85 years; and (3) patients without distant metastases of the tumor. The exclusion criteria were as follows: (1) pregnant patients or patients requiring emergency surgery; (2) patients who had not undergone intestinal anastomosis (such as Hartmann’s or Miles’s procedure); (3) patients who do not have the LCA; and (4) patients in whom LCA retention has been advised against because of a high metastasis risk of the root lymph nodes of the IMA confirmed intraoperatively. This was a controlled before-and-after study, and overall, 40 patients were selected from among the patients admitted for surgical treatment according to the inclusion and exclusion criteria. The investigators explained the purpose, significance, process, precautions, and other relevant issues pertaining to the study to the patients, and the patient decided on whether to participate in the study of their own will. The patients enrolled provided signed informed consent.

### Method

#### Intraoperative method

**Fig. 1:**
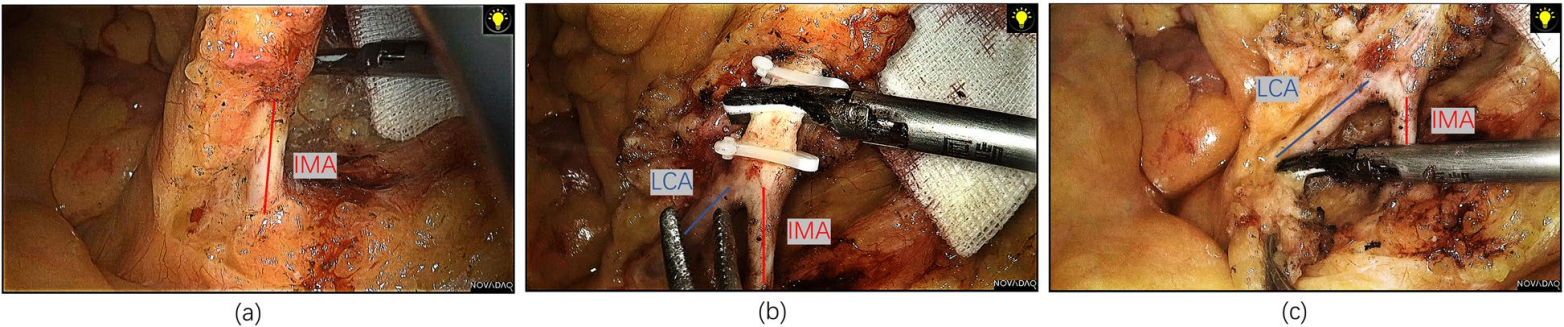
Radical resection with LCA retention was performed in patients with sigmoid or rectal cancer who participated in this study. **a** Exposing the root of the IMA. **b** Exposing the branch arteries of the IMA and ligating the IMA branch arteries except for the LCA. **c** Removing the adipose tissue around the IMA root to ensure the clearance of the lymph nodes

**Fig. 2:**
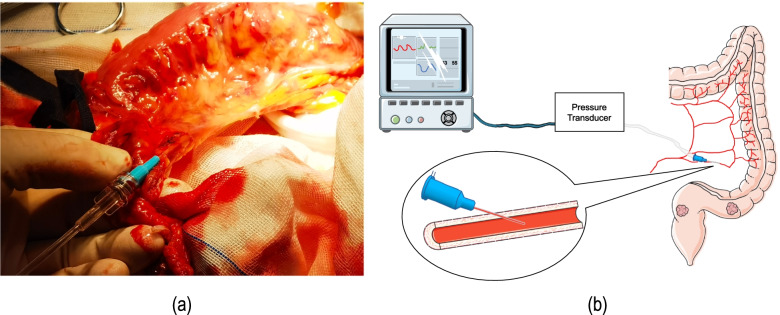
**a** Measurement of the MASP in the anastomotic colon during surgery. **b** A simple schematic diagram of the measurement of MASP

In laparoscopic surgery for sigmoid or rectal cancer, after carefully dissecting the IMA root (Fig. 1a), the artery wall was exposed to the bifurcation of the LCA and the superior rectal artery (Fig. 1b). After low ligation of the IMA, the adipose tissue around the IMA root was removed to ensure the clearance of the lymph nodes (Fig. 1c). Then, dissection was performed along the LCA to expose the IMV root and ligate it. A sterile silk thread was cut to the same length as the distance from the root of the IMA to the root of the LCA in the abdominal cavity and was measured out of the abdominal cavity. Finally, the intestinal part at the distal end of the tumor was cut off in the case of a macroscopically negative surgical margin. Invasive pressure measurement was performed at the marginal arterial arch of the pre-anastomosis area (Fig. 2). Meanwhile, systemic pressure (SP) measurement was performed on the forearm to calculate the mean pressure for further analysis. The marginal artery was ligated after a vascular indwelling needle was inserted, and the tumor was then resected. The colorectal/colo-anal tension-free anastomosis was completed using a circular stapler, and a barbed suture was used to reinforce the anastomosis. Indocyanine green (ICG) fluorescence imaging was performed to assess anastomotic tissue perfusion. Finally, the pelvis was filled with warm saline, and 50 ml of air was injected into the rectum through the anus to confirm the integrity of the anastomosis. Whether protective loop ileostomy should be performed in patients with colorectal cancer was determined based on intraoperative conditions.

#### Measurement of the MASP in the anastomotic colon

Heparin saline (500 mL of 5 U/mL) was placed into a pressurized bag and connected to a pressure sensor. Then, the vital signs monitor was connected to the pressure sensor and the arterial pressure mode was used. Then, the pressure sensor was emptied of air, placed at the same height as the right atrium, and zeroed. The vascular indwelling needle was placed in the edge arterial arch, fixed, and connected to the pressure sensor once the blood flowed out. MASP was recorded when the pressure stabilized. After the LCA was clipped, the MASP was re-recorded after the pressure stabilized.

#### Measurement of LCA diameter

The LCA diameter was measured using enhanced computed tomography of the abdomen within 3 cm after it was given off. To reduce measurement bias, two researchers who chose not to participate in the surgeries measured the LCA diameter before surgery in patients with colorectal cancer. If the difference in the measurements was less than or equal to 0.2 mm, the average of the measurements was considered as the patient’s LCA diameter. If, on the other hand, the difference was greater than 0.2 mm, a third researcher who was not involved in surgeries was selected to measure the LCA diameter, and the average of the two most similar values was considered as the patient’s LCA diameter.

### Statistical analysis

The independent samples *t* test was used to analyze the effect of clinically relevant factors on the MASP in the anastomotic colon before and after LCA clipping and the amplitude of MASP change. The paired samples *t* test was used to analyze the differences in anastomotic blood supply when the LCA was or was not retained and assess independent factors in subgroup that influence the change magnitude of MASP after LCA clamping. The independent predictors that influence the change magnitude of MASP after LCA clamping were identified using linear regression. Pearson correlation analysis was performed to evaluate the relationship between clinically relevant factors and the MASP in the anastomotic colon and explore the relationship between LCA diameter and the amplitude of MASP change after LCA clamping. Considering the degree of blood pressure decrease after LCA clamping as an effect evaluation, an increase in blood pressure greater than 10 mmHg (the median amplitude of MASP change) after LCA opening was regarded as a good improvement in anastomotic blood supply. Receiver operating characteristic (ROC) curve was used to determine the critical value of the minimum LCA diameter for retaining LCA with high sensitivity and specificity. Statistical analysis was performed using SPSS software (IBM SPSS 26.0; SPSS Inc., Armonk, NY). The significance level was set at a *p* value of less than 0.05.

## Results

### General results of surgery

In all, 40 patients participated in the study, including 26 and 14 patients with rectal and sigmoid colon cancer, respectively, of which 6 had partial intestinal obstruction before surgery. Eighteen patients underwent protective ileostomy. One patient developed an anastomotic abscess. Each anastomosis was tension free, and no patient required mobilization of the splenic flexure. During surgery, the marginal arteries were well protected. Patients’ anastomosed colons did not appear to have macroscopic ischemic changes under the near-infrared system.

### Identification of clinical factors affecting the MASP in the anastomotic colon when the LCA was clamped and open and the amplitude of MASP change

**Fig. 3:**
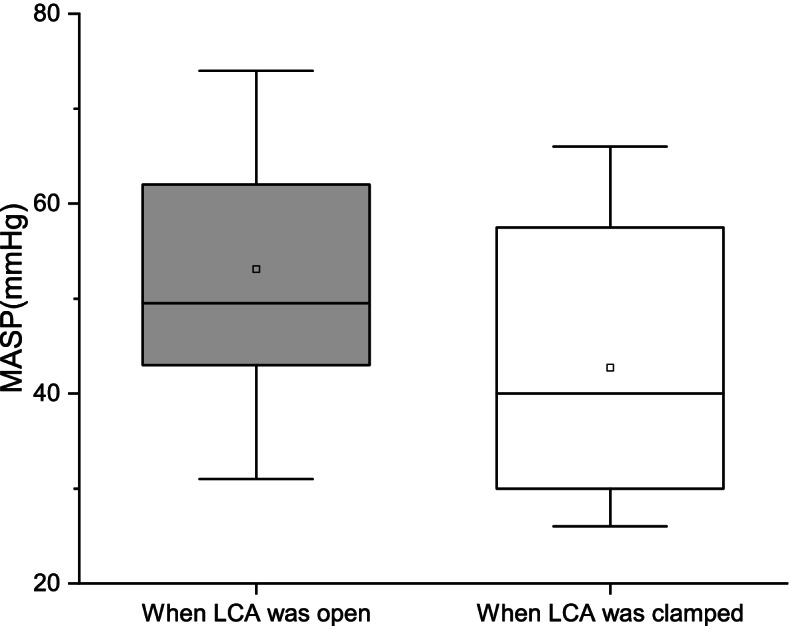
MASPs in patients with sigmoid or rectal cancer when the LCA was clamped and open. The MASPs when the LCA was open and when it was clamped were 53.1 ± 12.38 and 42.76 ± 12.71, respectively. There were significant differences between the two values (*p* < 0.001)

All participants underwent radical resection for sigmoid or rectal cancer with LCA retention. Furthermore, the MASP in the anastomotic colon was measured when the LCA was clamped and open in every patient. The mean MASP (± standard error) when the LCA was clamped was 53.10 ± 12.38 mmHg, while that when the LCA was open was 42.73 ± 12.71; there was a significant difference between the two values (*p* < 0.001) (Fig. 3). And the mean amplitude of MASP change is 10.38 ± 6.51 mmHg (median 10 mmHg). The MASP in the anastomotic colon when the LCA was clamped and open and the amplitude of MASP change among all patients were analyzed separately with respect to sex, age, diabetes, hypertension, body mass index (BMI), arterial calcification, SP, LCA diameter, and distance from the root of the LCA to the root of the IMA (Table 1). Univariate analysis revealed that sex, body mass index, and arterial calcification were potential predictive factors for the MASP in the anastomotic colon regardless of whether the LCA was clamped or open, and SP mainly affected the MASP in the anastomotic colon when the LCA was open (*p* = 0.024). In the subgroups of patients with SP < 80 mmHg, hypertension, diabetes and LCA diameter < 2 mm, the MASP when LCA was open showed no statistical differences compared with the MASP when LCA was clamped (Table 2). In addition, Table 3 shows that the amplitude of MASP change after LCA clamping was significantly greater in patients with an LCA diameter of ≥ 2 mm than in those with an LCA diameter of < 2 mm (15.5 mm vs 4.1 mm, *p* < 0.001), which indicated that LCA diameter was an independent predictor that influence the change magnitude of MASP after LCA clamping.

**Table 1:** Assessment of independent predictors that influence the MASPs with LCA opening and clamping

Variables	Patients	MASP when LCA was open (mmHg)	*P* value	MASP when LCA was clamped (mmHg)	*P* value
Sex					
Male	22	48.23	**0.004**	36.45	**< 0.001**
Female	18	59.06		50.39	
Age (years)					
≥ 60	18	52.28	**0.709**	40.44	**0.311**
< 60	22	53.77		44.59	
BMI					
≥ 25	20	60.5	**< 0.001**	55.20	**< 0.001**
< 25	20	45.7		33.25	
Hypertension					
No	31	53.32	**0.836**	41.84	**0.42**
Yes	9	52.33		45.78	
Diabetes					
No	37	53.16	**0.913**	42.38	**0.552**
Yes	3	52.33		47.00	
Arterial calcification					
No	17	61.29	**< 0.001**	48.65	**0.017**
Yes	23	47.04		38.34	
SP (mmHg)					
≥ 80	36	54.56	**0.024**	43.81	**0.108**
< 80	4	40.00		33.00	
The distance from the root of the LCA to the root of the IMA (cm)					
≥ 3.0	25	50.68	**0.150**	40.80	**0.221**
< 3.0	15	57.13		45.93	
LCA diameter (mm)					
≥ 2.0	22	55.82	**0.126**	40.31	**0.178**
< 2.0	18	49.78		45.67	
Anastomotic complications					
No	39	53.44	**0.290**	43.13	**0.215**
Yes	1	40.00		27.00

**Table 2:** Assessment of independent factors in subgroup that influence the change magnitude of MASP after LCA clamping

Variables	Patients	MASP when LCA was open (mmHg)	MASP when LCA was clamped (mmHg)	*P* value
Sex				
Male	22	48.23 ± 10.33	36.45 ± 8.79	**<0.001**
Female	18	59.06 ± 12.31	50.39 ± 12.74	**0.046**
Age (years)				
≥ 60	18	52.28 ± 14.00	40.44 ± 12.54	**0.012**
< 60	22	53.77 ± 11.18	44.59 ± 12.84	**0.015**
BMI				
≥ 25	20	60.50 ± 11.96	52.20±10.06	**0.023**
< 25	20	45.70 ± 7.51	33.25 ± 6.46	**<0.001**
Hypertension				
No	31	53.32 ± 12.73	41.84 ± 12.78	**<0.001**
Yes	9	52.33 ± 11.80	45.78 ± 12.73	**0.274**
Diabetes				
No	37	53.16 ± 12.18	42.38 ± 12.86	**<0.001**
Yes	3	52.33 ± 17.90	47.00 ± 12.12	**0.691**
Arterial calcification				
No	17	61.29 ± 10.29	48.65 ± 14.52	**0.004**
Yes	23	47.04 ± 10.23	38.35 ± 9.28	**0.006**
SP (mmHg)				
≥ 80	36	54.56 ± 12.21	43.81 ± 12.80	**< 0.001**
< 80	4	40.00 ± 0.82	33.00 ± 6.98	**0.138**
The distance from the root of the LCA to the root of the IMA (cm)				
≥ 3.0	25	50.68 ± 10.28	40.80 ± 12.36	**0.003**
< 3.0	15	57.13 ± 14.77	45.93 ± 13.07	**0.036**
LCA diameter (mm)				
≥ 2.0	22	55.82 ± 12.70	40.32 ± 13.94	**< 0.001**
< 2.0	18	49.78 ± 11.46	45.67 ± 10.68	**0.273**
Anastomotic complications				
No	39	53.44 ± 12.36	43.13 ± 12.62	**< 0.001**
Yes	1	40	27	**–**

**Table 3:** Assessment of independent predictors that influence the change magnitude of MASP after LCA clamping

Variables	Patients	The amplitude of MASP change (mmHg)	Univariate *P* value	Multivariate *P* value
Sex				
Male	22	11.77 ± 8.83	**0.239**	**0.272**
Female	18	8.67 ± 7.29		
Age (years)				
≥ 60	18	11.83 ± 8.40	**0.316**	**0.747**
< 60	22	9.18 ± 8.06		
BMI				
≥ 25	20	5.30 ± 8.12	**0.111**	**0.126**
< 25	20	12.45 ± 7.98		
Hypertension				
No	31	11.48 ± 8.81	**0.114**	**0.693**
Yes	9	6.56 ± 4.13		
Diabetes				
No	37	10.78 ± 8.31	**0.275**	**0.558**
Yes	3	5.33 ± 5.77		
Arterial calcification				
No	17	12.65 ± 7.77	**0.135**	**0.589**
Yes	23	8.70 ± 8.30		
SP (mmHg)				
≥ 80	36	10.75 ± 8.35	**0.394**	**0.085**
< 80	4	7.00 ± 6.93		
The distance from the root of the LCA to the root of the IMA (cm)				
≥ 3.0	25	9.88 ± 8.16	**0.629**	**0.134**
< 3.0	15	11.20 ± 8.55		
LCA diameter (mm)				
≥ 2.0	22	15.50 ± 7.40	**< 0.001**	**< 0.001**
< 2.0	18	4.1 ± 3.43		

### Relationship between MASP and clinical factors

**Fig. 4:**
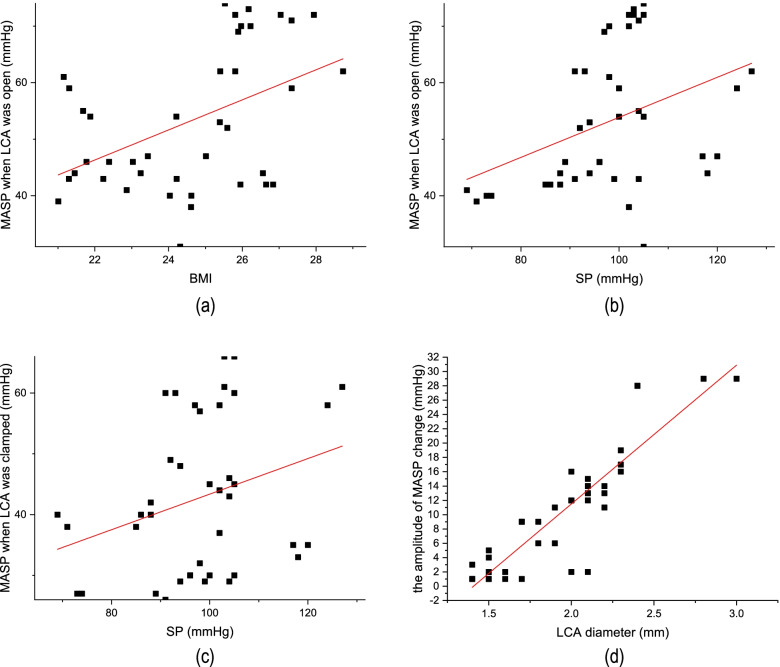
Scatter diagram of clinical factors associated with MASPs. **a** Scatter diagram of BMI and MASP when the LCA was open. The black dots represent the corresponding BMI and MASP when the LCA was open, and the red line represents the linear fitting (*r* = 0.460). **b**, **c** Scatter diagram of BMI and MASP when the LCA was open and when it was clamped. The black dots represent the corresponding SP and MASP, and the red line represents the linear fitting (*r* = 0.376 and 0.302). **d** Scatter diagram of the LCA diameter and the amplitude of MASP change after the LCA was clamped. The black dots represent the corresponding LCA diameter and the amplitude of MASP change, and the red line represents the linear fitting (*r* = 0.891)

Additional analysis conducted based on the univariate analysis showed that SP, BMI, and LCA diameter were the factors potentially influencing MASP. A positive linear correlation was found between BMI and MASP in the anastomotic colon when the LCA was open (Fig. 4a) as well as between SP and MASP in the anastomotic colon regardless of whether the LCA was clamped or open (Fig. 4b, c). The analysis also revealed a strong positive linear relationship between LCA diameter and the amplitude of MASP change (*r* = 0.891) (Fig. 4d).

### Threshold values of the LCA inner diameter affecting the anastomotic blood supply

A MASP change amplitude of ≥ 10 mmHg after LCA clamping was considered to have a large impact on the blood supply in the anastomotic area. Further, ROC analysis was performed to determine the LCA threshold diameter that would predict a significant effect on the anastomotic blood supply. LCA diameter was found to be a valuable predictive test factor for whether the LCA can affect the anastomotic blood supply (AUC = 0.971, Figure S1). When the LCA diameter was equal to 1.95 mm, the Youden index was the largest (Youden index = 0.847) (Table S1). This indicates means that an LCA diameter of 1.95 mm is the optimal threshold for predicting whether the LCA can significantly affect the anastomotic blood supply.

## Discussion

IMA branches vary greatly between individuals. Therefore, radical resection for sigmoid or rectal cancer with LCA retention is relatively complex and has a relatively long duration of operation compared with procedures that do not involve LCA retention. In addition, the National Comprehensive Cancer Network guidelines did not specify whether the LCA should be preserved during surgery for rectal or sigmoid colon cancer [19]. Therefore, most surgeons prefer high ligation of the IMA. The current controversy mainly focuses on whether LCA retention can reduce the incidence of anastomotic complications, that is, whether it can improve the intestinal blood supply in the anastomotic area. In our study, we used the MASP in the anastomotic colon as an index to evaluate the anastomotic blood supply more intuitively. However, needle insertion into the marginal artery arch of the anastomotic colon is complicated and requires surgical expertise. Therefore, hopefully, a simpler, more effective, and intuitive observation index of the anastomotic blood supply can be found in the future.

Studies have shown that the arterial stiffness index is significantly higher in obese patients than in healthy individuals. Therefore, the blood pressure in obese patients is also higher than that in non-obese individuals [20], which is consistent with our findings. But interestingly, obesity is a known risk factor for anastomotic leakage, which seems to contradict our findings that anastomotic blood supply is superior in obese patients to non-obese patients. A possible explanation is that obesity is associated with the chronic pro-inflammatory state which may impair normal tissue repair and impact anastomotic healing [21]. Moreover, there is usually a thickened mesentery or a disproportionately large omentum in obese patient. These factors might increase the technical difficulty of colonic anastomosis formation and the rate of anastomotic leakage. And the technical difficulty might further increase with the lowest of rectal tumors [22]. In addition, the magnitude of MASP changes after LCA clipping in obese patients was significantly lower than that in non-obese patients (5.3 mmHg vs 12.45 mmHg). Although there was no statistical difference in the magnitudes (*p* = 0.111), larger sample sizes in later studies are expected to eliminate systematic errors. The MASP was significantly lower in male than in female. This is consistent with the finding that male gender is a known risk factor for leakage [8]. Furthermore, the degree of arterial stiffness in young women has been found to be lower than that in age-matched men; this sex difference in arterial stiffness can be reversed with age [23]. This also explains why the MASP in female patients in our study was significantly higher than in male patients.

There was a positive linear relationship between patients’ systemic blood pressure and their MASP; this finding is consistent with those of Guo et al. [24].

By comparing MASPs when LCA is in different states, we found that the overall MASP when the LCA was open was significantly higher than that when the LCA was clamped. These data verify that LCA retention is effective in improving blood supply to the anastomosis area. In addition, our findings confirmed a positive correlation between the amplitude of MASP change in the anastomotic colon after LCA clamping and the LCA diameter, in that the larger the LCA diameter, the drop in the MASP in the anastomotic colon after LCA clamping was greater than that before LCA clamping. Therefore, colorectal cancer patients with larger LCA diameters also derive greater benefits from intraoperative preservation of the LCA. For patients with LCA stenosis, the middle colic artery is the dominant blood vessel that supplies blood to the left colon, and the LCA has little effect on the blood supply to the left colon. Therefore, retention of the LCA is of little significance in these patients. The middle colic artery is an important branch of the superior mesenteric artery (SMA) and an important source of blood supply to the marginal artery. The middle colic arteries are missing in 3–5% of patients [25]. In these patients, radical resection of the sigmoid colon or rectal cancer with high IMA ligation is likely to result in insufficient blood supply in the anastomosis area, and preserving the LCA artery is the ideal surgical option.

There was a large variation in the track of the LCA: in more than half of the patients, the LCA are able to reach the splenic flexure, and approximately 20% of the patients, the LCA only reached the lower section of the descending colon or even lacked [18, 26]. This implies that the vast majority of the left colonic blood flow is affected by the LCA. However, no study has confirmed the relationship between the track of the LCA and the blood supply in the anastomosis area. This may be a direction for exploring indications for LCA retention.

There are some limitations of this study. Firstly, the sample size of this study was small, with only 40 patients included in the study. As a result, we were not able to perform external validation of our results. Secondly, the variables included in this study may be inadequate, such as the track of the LCA, which may have an effect on MASP of anastomotic colon and should be included in future research. What’s more, this study only included patients with colorectal tumors 5–25 cm from the anus. This may not reflect the effect of LCA on blood supply of anastomotic colon when tumor location is biased toward descending colon.

## Conclusion

Taken together, our results indicate that retaining LCA did effectively enhance the anastomotic blood supply. Furthermore, LCA diameter was an important factor affecting blood supply in the anastomosis area, and a lager LCA diameter seemed a greater effect the LCA had on the blood supply in the anastomotic area. While the LCA root location was not related to the anastomotic blood supply.

## Supplementary Information

**Additional file 1: Figure S1.** ROC curve for the LCA diameter in predicting the ability of the LCA to affect the anastomotic blood supply.

**Additional file 2: Table S1.** ROC curve coordinates of LCA diameter.

## Data Availability

The datasets used and analyzed during the current study are available from the corresponding authors on reasonable request.

## Abbreviations

LCA: Left colic artery
IMA: Inferior mesenteric artery
MASP: Marginal artery stump pressure
SP: Systemic pressure
ICG: Indocyanine green
ROC: Receiver operating characteristic
SMA: Superior mesenteric artery
