# Supplementary figures and images for: Left colic artery diameter is an important factor affecting anastomotic blood supply in sigmoid colon cancer or rectal cancer surgery: a pilot study
Source: World J Surg Oncol. 2022 Sep 27;20:313. doi: 10.1186/s12957-022-02774-0 (PMC9513983; doi:10.1186/s12957-022-02774-0)

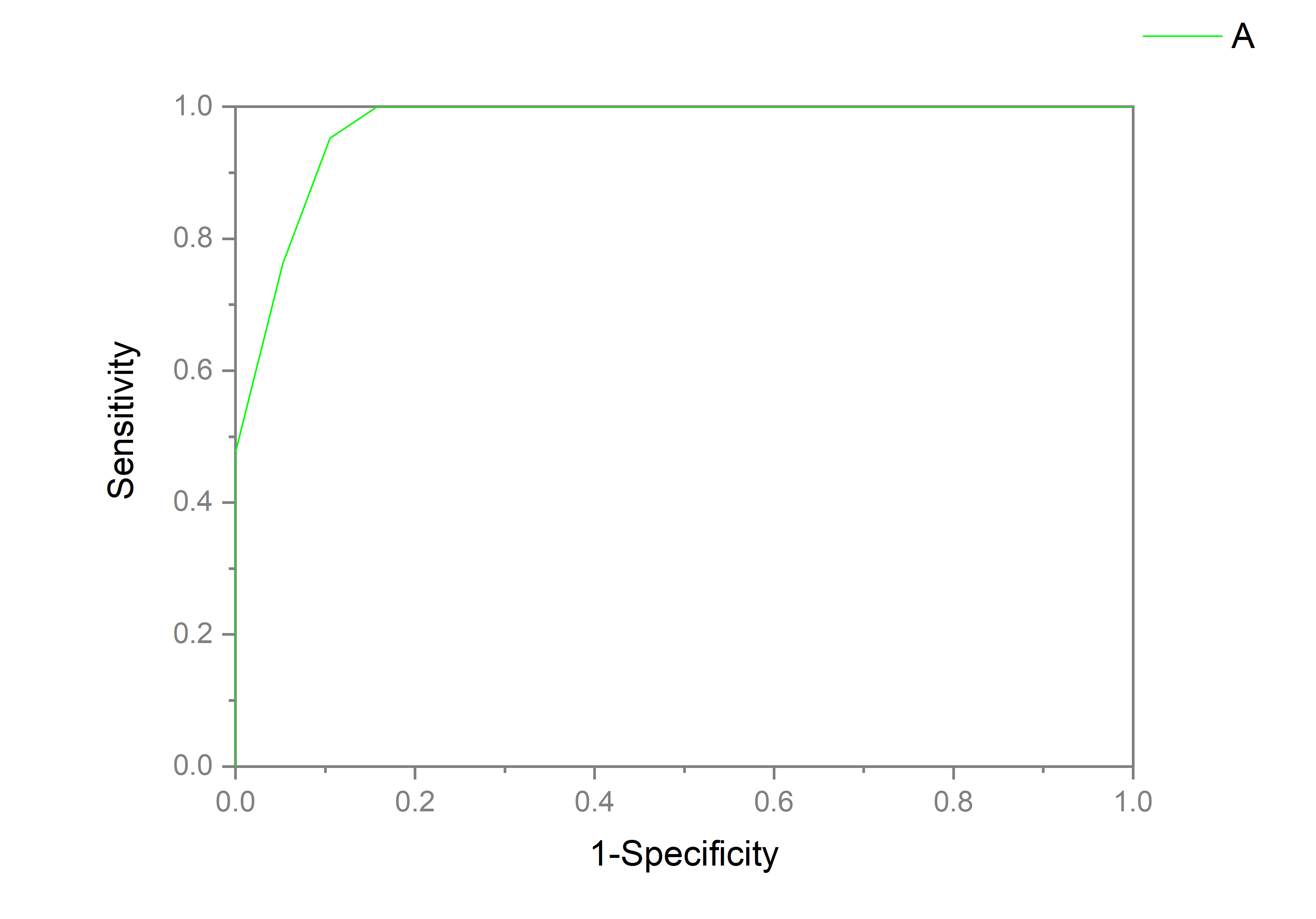

Supplement: Supplementary file 1 — Additional file 1: Figure S1. ROC curve for the LCA diameter in predicting the ability of the LCA to affect the anastomotic blood supply. [file 12957_2022_2774_MOESM1_ESM.jpg]
